# Renal denervation improves cardiac function independently of afterload and restores myocardial norepinephrine levels in a rodent heart failure model

**DOI:** 10.1038/s41440-024-01580-3

**Published:** 2024-02-02

**Authors:** Matúš Miklovič, Olga Gawryś, Zuzana Honetschlägerová, Petr Kala, Zuzana Husková, Soňa Kikerlová, Zdeňka Vaňourková, Šárka Jíchová, Alena Kvasilová, Misuzu Kitamoto, Hana Maxová, Guillermo Puertas-Frias, Tomáš Mráček, David Sedmera, Vojtěch Melenovský

**Affiliations:** 1 Center for Experimental Medicine, Institute for Clinical and Experimental Medicine—IKEM, Prague, Czech Republic; 2 Department of Pathophysiology, 2nd Faculty of Medicine, Charles University, Prague, Czech Republic; 3 Department of Cardiology, University Hospital Motol and 2nd Faculty of Medicine, Charles University, Prague, Czech Republic; 4 Institute of Anatomy, First Faculty of Medicine, Charles University, Prague, Czech Republic; 5 Institute of Physiology, Czech Academy of Sciences, Prague, Czech Republic; 6 Department of Cardiology, Institute for Clinical and Experimental Medicine—IKEM, Prague, Czech Republic

**Keywords:** Heart failure, Norepinephrine, Renal denervation, Volume overload, Sympathetic nervous system

## Abstract

Renal nerves play a critical role in cardiorenal interactions. Renal denervation (RDN) improved survival in some experimental heart failure (HF) models. It is not known whether these favorable effects are indirect, explainable by a decrease in vascular afterload, or diminished neurohumoral response in the kidneys, or whether RDN procedure per se has direct myocardial effects in the failing heart. To elucidate mechanisms how RDN affects failing heart, we studied load-independent indexes of ventricular function, gene markers of myocardial remodeling, and cardiac sympathetic signaling in HF, induced by chronic volume overload (aorto-caval fistula, ACF) of Ren2 transgenic rats. Volume overload by ACF led to left ventricular (LV) hypertrophy and dysfunction, myocardial remodeling (upregulated Nppa, MYH 7/6 genes), increased renal and circulating norepinephrine (NE), reduced myocardial NE content, increased monoaminoxidase A (MAO-A), ROS production and decreased tyrosine hydroxylase (+) nerve staining. RDN in HF animals decreased congestion in the lungs and the liver, improved load-independent cardiac function (Ees, PRSW, Ees/Ea ratio), without affecting arterial elastance or LV pressure, reduced adverse myocardial remodeling (Myh 7/6, collagen I/III ratio), decreased myocardial MAO-A and inhibited renal neprilysin activity. RDN increased myocardial expression of acetylcholinesterase (Ache) and muscarinic receptors (Chrm2), decreased circulating and renal NE, but increased myocardial NE content, restoring so autonomic control of the heart. These changes likely explain improvements in survival after RDN in this model. The results suggest that RDN has remote, load-independent and favorable intrinsic myocardial effects in the failing heart. RDN therefore could be a useful therapeutic strategy in HF.

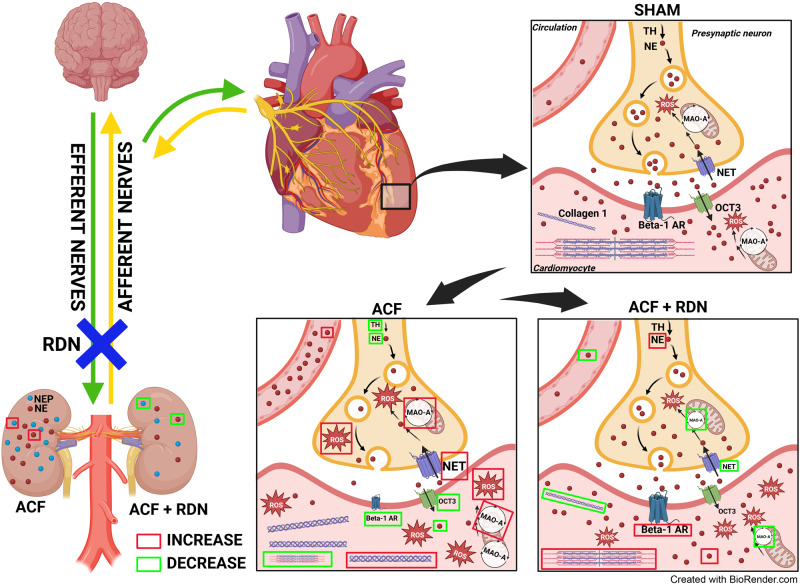

## Introduction

Renal sympathetic innervation plays an important role in cardiorenal interactions and in the pathophysiology of heart failure (HF) [1, 2]. The renal nerves contain both afferent and efferent sympathetic nerve fibers, establishing a bidirectional link between the kidneys and the brain [3]. Through this connection, the brain centers, located in the hypothalamus and in the brain stem, collect renal and other sensory inputs and regulate sympathetic nerve output to peripheral organs, including the kidneys and the heart [1]. Chronic HF is accompanied by sympathetic overactivation, increased norepinephrine (NE) release from the heart and the kidneys into the plasma [4], depleted NE in the myocardium [5], increased production of reactive oxygen species (ROS) [6], and downregulated beta-1 adrenergic receptors (Adrb1), which all contribute to decreased contractility, fibrosis and further propagation of myocardial damage [7–9].

Renal denervation (RDN) is a non-pharmacological treatment method consisting of the targeted destruction of nerve fibers around the renal arteries by radiofrequency energy, ultrasound or by locally-applied chemical toxin [10–14], that eliminates both efferent and afferent sympathetic signals, reduces kidney NE and attenuates the central sympathetic drive [15, 16]. RDN demonstrated marked antihypertensive effects in experimental studies and also in clinical trials [17].

Intriguingly, RDN showed favorable effects not only in hypertension but more recently also in experimental models of HF, where it improved survival [10, 12, 18, 19]. This is somewhat surprising because pharmacological unselective central sympathetic inhibition in HF patients led to excessive mortality and was abandoned [20]. The mechanisms of favorable effects of RDN in HF have not yet been sufficiently elucidated. The key unresolved questions remain, whether RDN effects in HF are explainable by an attenuated neurohumoral response in the kidneys, by reduced vascular afterload, or whether RDN procedure per se has direct myocardial effects in the failing heart.

In this study, we investigated the effects of RDN on HF due to volume overload from aortocaval fistula (ACF)—well established HF model of high cardiac output with markedly expressed neurohumoral activation and congestion [5, 21, 22]. We used Ren-2 transgenic rat (TGR) [19, 23, 24], a strain that displays rapid and pronounced onset of HF symptoms after ACF induction [19]. It is known that in TGR rats, a hypotensive effect occurs in very early stages after ACF, due to the transfer of volume and pressure from the aorta to the inferior vena cava [19, 25]. To separate the impact of RDN on vascular load from intrinsic myocardial effects, we utilized pressure-volume (PV) analysis, and we studied the effects on molecular markers of myocardial remodeling. We further analyzed in a greater detail changes in the myocardial sympathetic system, that are grossly deregulated in this HF model [5, 26].

## Methods

### Animals and the protocol

The study was performed in accordance with relevant regulations and was approved by the Animal Ethics Committee of IKEM and by the Ministry of Health of the Czech Republic (#12468/2021-5/OVZ). Heterozygous TGR were generated by breeding female homozygous HanSD rats with male homozygous TGR as described [27]. Eight-week-old TGR underwent ACF surgery to induce volume overload. After 1 week, animals underwent bilateral RDN (mechanical and chemical by topical phenol application) from laparotomy [28, 29]. Two weeks after RDN, the functions of the left ventricle (LV) were measured by PV analysis, and the animals were examined using echocardiography each week. Animals were killed by thiopental sodium i.p. overdose (in the case of in vivo measurements of echocardiography and PV analysis), or by decapitation (hormonal and molecular analyses).

### Aortocaval fistula and renal denervation surgery

The animals were anaesthetized using ketamine and midazolam (Calypsol, Gedeon Richter, Hungary, 160 mg/kg and Midazolam, Kalcex, Latvia, 160 mg/kg, i.p.). The ACF was created as described previously and this procedure is routinely performed in our laboratory [19, 21, 30]. The sham-operated rats underwent opening and closing of the abdominal cavity, without the aortocaval fistula procedure. After the surgery, meloxicam analgesia (1–2 mg/kg/day, s.c.) was given for 2–3 days.

Bilateral RDN procedure was performed 1 week after ACF surgery. Animals were anaesthetized using ketamine/midazolam and bilateral RDN procedure was performed as described in previous studies [19, 31–33]. Intact animals underwent laparotomy and retraction of the abdominal organs.

### Echocardiography

An echocardiographic examination was performed on the day of surgery before the creation of ACF/sham. The second examination was performed after 1 week, before RDN/intact surgery, and once per week during the next 2 weeks until the end of the experiment. Prior to the echocardiographic examination, the animals were anesthetized with 4% isoflurane (IsoVet®, Piramal Healthcare, UK). During the image acquisition, the rats were maintained under isoflurane anesthesia (1.5–2%, Combi-vet® system, Rothacher Medical GmbH, Heitenried, Switzerland). B-Mode and M-Mode images were recorded in parasternal long and short axis view and used for measurements of dimensions of LV internal diameter, and anterior and posterior walls. Echocardiographic examination was done by Vevo® 2100 Imaging System with the MS250S transducer (13–24 MHz), and evaluated in VevoLab (v3.2.0, FUJIFILM VisualSonics, Inc., Toronto, ON, Canada).

### Pressure-volume analysis

Rats were anesthetized with thiopental (50 mg/kg, i.p., VAUB Pharma a.s., Roztoky, CZ) and echocardiography was performed as described above. Rats were intubated and artificially ventilated through the procedure (Ugo Basile, Gemonio, IT). The left jugular vein was cannulated with saline for securing central venous access. A balloon catheter (LeMaitre Single Lumen Embolectomy Catheter, 2F, Burlington, MA, USA) was inserted via the left femoral vein to the vena cava inferior, below the diaphragm to maintain the best position for preload reduction [34]. Functions of the LV were invasively assessed by a PV catheter (Millar, 2F, Houston, TX, USA) introduced into the LV via the right carotid artery as described in previous studies [35–37]. Volume signal was calibrated by cuvette calibration unit of known volumes fulfilled with heparinized warm blood taken from LV after the experiment. Data from PV loops were captured and analyzed in LabChart Pro software (ADInstruments, Bella Vista, NSW, Australia) as discussed in detail in our previous study [34].

### Gene expression analysis

LV was homogenized in RNAzol® RT (#RN190; Molecular Research Center, Inc., Cincinnati, USA) and RNA free water was added to samples and mixed. After the precipitation the samples were centrifuged (12,000 × *g*, 15 min, 4 °C) and the supernatant was transferred. The RNA was precipitated with 75% ethanol and gently mixed, incubated for 15 min, room temperature (rt), centrifuged (12,000 × *g*, 20 min, 4 °C) and the supernatant was discarded. The pellet was washed twice with 75% ethanol, centrifuged (7500 × *g*, 5 min, 4 °C) and dried in a thermoblock at 55 °C. The pellet was dissolved in RNA free water, incubated in the thermoblock for 10 min and stored at −80 °C until analysis. The concentration and purity of the RNA were measured. Bio-Rad C1000 Thermal cycler (Bio-Rad s.r.o., Prague, Czech Republic) was used with High Capacity cDNA Reverse Transcription Kit (#4368813; Applied Biosystems, Foster City, CA, USA). Transcribed cDNA samples were diluted with DEPC-treated water and mixed with TaqMan Fast Advanced Master Mix (#4444556; Applied Biosystems, Foster City, CA, USA). The solution was transferred to a TaqMan Array Card (384-well microfluidics TaqMan array cards; custom setting of selected genes), centrifuged twice (1200 RPM, 1 min, 4 °C) and sealed. The quantification was done on ViiA™ 7 Real-time PCR system (Applied Biosystems, Foster City, CA, USA) and the measurement of mRNA expressions was performed in accordance with the manufacturer’s instructions.

Relative gene expression was calculated by the 2*−*∆∆Ct method, which is frequently used for such experiments [38–40]. Housekeeping gene GAPDH was used as the normalizer to calculate relative gene expression. Final results were expressed as the n-fold difference in gene expression of mRNA of target genes between experimental and control.

### Immunohistochemistry

Dissected basal halves of free walls of the LV were whole-mount stained with anti-tyrosine hydroxylase primary antibody (1:500, rabbit AB152, Merck Millipore) visualized with Cy5 coupled goat-anti-rabbit secondary antibody (1:200, Jackson Immuno Research) as described [41]. Incubation times were prolonged (blocking 24 h in normal goat serum, 1:10, primary antibody 48 h, washing 12 h, secondary antibody 24 h, all at rt with continuous gentle rocking), and Triton-X concentration was increased at 0.1%.

After staining, the samples were pinned epicardial side up to the bottom of a deep Petri dish covered with Sylgard and cleared in Scale2 for at least 48 h [42]. The samples were then examined on a confocal Olympus BX61 system (FluoView 1000) using a 2x, 0.14 NA dry objective (overview picture) followed by 25x ScaleView objective (NA 1.0). Three confocal stacks spanning at least 50-micron depth were collected from different location at 1-micron z-step. For analysis, sub-stacks of 10 µm thickness were selected from the subepicardial region, and maximum intensity projections of green channel (488 nm excitation, detecting tissue autofluorescence derived mostly from the myocytes) and far-red channel (635 nm excitation, specific fluorescence of anti-tyrosine hydroxylase immunostaining) were created in ImageJ. Percentage of red to red + green channel was then calculated after signal thresholding in ImageJ.

### Myocardial ROS production by monoamine oxidase A

Myocardial hydrogen peroxide production by monoamine oxidase A (MAO-A) was determined fluorometrically measuring the oxidation of Amplex Red reagent (Thermo Fisher Scientific, Waltham, MA, USA) coupled to the enzymatic reduction of H_2_O_2_ by horseradish peroxidase. Briefly, 10% homogenates were prepared in ice-cold KCl-based media (120 mM KCl, 3 mM HEPES, 5 mM KH2PO4, 3 mM MgSO4, and 1 mM EGTA, pH 7.2) from frozen free wall of LV using zirconium oxide grinding balls (3 min, 30 Hz), Retsch MM 400 mixer mill (Retsch, Haan, Germany) and filtered through a fine mesh. Protein concentration was estimated by the BCA method. An aliquot of the homogenate was saved for SDS-PAGE analyses. The assay was performed using 0.15 mg/ml of protein in KCl-based media with or without the MAO-A substrate tyramine (50 μM) or its specific inhibitor clorgylin (1 μM), in the presence of 50 μM Amplex Red and 1 U/ml HPR. A standard curve of 1–10 μM H_2_O_2_ was established in every plate to calibrate the signal to nmol produced. The increase in the fluorescence was recorded for 30 min at 37 °C with an Infinite M200 plate reader (Tecan Group Ltd., Männedorf, Switzerland) at 544/590 nm.

### Western blot analysis

LV tissue (free wall) was homogenized and protein concentration in the supernatant was measured using Pierce BCA protein assay (Thermo Fisher Scientific, Waltham, MA, USA). Protein was separated by sodium dodecyl sulfate polyacrylamide gel electrophoresis (SDS-PAGE) and transferred onto the polyvinyl difluoride membrane in transfer buffer at 100 V for 1.5 h. Membranes were blocked with 2.5% BSA/5% non-fat dry milk in TRIS buffered saline with Tween20 (TBS-T) and washed with TBS-T. The membranes were incubated with primary antibodies overnight at 4 °C (more antibody dilutions details in supplement). After incubation and washing, the membranes were incubated with horseradish peroxidase-conjugated secondary antibody for 1 h at rt. After last washing, the immunoblots were exposed to SuperSignal West Dura Substrate (Thermo Scientific, Rockford, IL, USA) for chemiluminescent detection. Relative densitometry was determined using ImageJ software (NIH, Bethesda, MD, USA). Protein data were normalized to the housekeeping protein GAPDH. Final results were expressed as the n-fold difference in target protein expression between experimental and control group.

### Neprilysin activity in kidney tissue

Neprilysin activity was assessed in whole kidney samples as described before [43, 44]. Briefly, kidney tissue was homogenized in ice-cold neprilysin assay buffer supplemented with protease inhibitors aprotinin and phenylmethylsulfonyl fluoride using mixer mill MM400, centrifuged (12,000 × *g*, 10 min, 4 °C) and supernatant was collected. Neprilysin activity was measured by a fluorometric assay (K487-100; BioVision, Milpitas, CA, USA).

### The measurement of norepinephrine in kidney and heart tissue

NE was measured in kidney cortex and LV heart samples. Briefly, wet tissue samples were weighted and homogenized in 0,05 M PB (pH 7.4) supplemented with protease inhibitor cocktail (Sigma-Aldrich, St. Louis, MO, USA) and ascorbic acid using mixer mill MM400, centrifuged twice (3000 × *g*, 10 min, 4 °C and 10,000 × *g*, 10 min, 4 °C) and supernatant was collected. NE concentration was measured by a solid phase enzyme-linked immunosorbent assay based on the sandwich principle, using commercially available ELISA kit (RE59261; IBL International, Hamburg, Germany). More methodological details are provided in Supplementary Information.

### Statistical analysis

Statistical analysis of the data was performed using Graph-Pad Prism software v9.4.1 (Graph Pad Software, San Diego, CA, USA). All results are presented as the mean ± standard error of the mean. The data were analyzed using one-way ANOVA (organ weights, core hemodynamic parameters, gene and protein expressions) with Fisher’s LSD post hoc test or two-way ANOVA (echocardiography results) followed by Tukey’s post hoc multiple comparison test. The values of *p* below 0.05 were considered as statistically significant.

## Results

### Weights, cardiac dimensions and principal LV hemodynamics

Table 1 shows organ weights and hemodynamics in the sham-operated control group, in a group with HF induced by ACF, and in a group with ACF and RDN. The sham/RDN group compared to the sham/intact group displayed no significant changes in organ weight parameters but significantly decreased end-systolic pressure (149 ± 3.8 vs. 169 ± 3.7 mmHg, *p* < 0.05) and mean LV pressure (65 ± 2.2 vs. 75.4 ± 3.7 mmHg, *p* < 0.05).

**Table 1:** Organ weights and principal hemodynamic parameters of the left ventricle

Strain	sham/intact	sham/RDN	ACF/intact	ACF/RDN
Organ weight (g)				
*n*	25	28	25	28
Body weight	438 ± 9.59	439 ± 8.25	425 ± 8.38	418 ± 7.2
Heart weight	1.62 ± 0.02	1.6 ± 0.03	2.24 ± 0.03*	1.95 ± 0.04*^†^
Left atrium	0.04 ± 0.001	0.041 ± 0.001	0.074 ± 0.002*	0.065 ± 0.002*^†^
Left ventricle (with IVS)	1.22 ± 0.02	1.19 ± 0.02	1.53 ± 0.02*	1.36 ± 0.03*^†^
Kidney weight	1.61 ± 0.03	1.65 ± 0.04	1.56 ± 0.04	1.51 ± 0.02
Lungs weight	1.81 ± 0.06	1.85 ± 0.04	2.47 ± 0.12*	2.1 ± 0.06*^†^
Liver weight	15.97 ± 0.43	15.49 ± 0.4	16.6 ± 0.39	14.83 ± 0.33^†^
Hemodynamics				
*n*	8	11	14	10
Stroke work (mmHg*µl)	6957 ± 784	7334 ± 646	10,757 ± 581*	7953 ± 841^†^
Cardiac output (μl/min)	17,305 ± 2052	20,426 ± 1193	32,205 ± 1843*	24,544 ± 2765^†^
Stroke volume (μl)	44.1 ± 4.8	49.1 ± 3.5	78.9 ± 3.6*	60.5 ± 6.6^†^
Mean ventricular pressure (mmHg)	75.4 ± 3.7	65 ± 2.2*	68.4 ± 2.3	67.1 ± 2.2
End-systolic pressure (mmHg)	169 ± 3.7	149 ± 3.8*	143 ± 3.1*	142 ± 2.2*
End-diastolic pressure (mmHg)	7 ± 0.78	5.5 ± 0.63	12.7 ± 1.63*	8.2 ± 0.69^†^
End-diastolic volume (µl)	230 ± 21.1	260 ± 13.3	356 ± 13.3*	264 ± 27^†^
End-systolic volume (µl)	175 ± 17.9	207 ± 6.7	260 ± 12.3*	191 ± 19.3^†^
Heart rate (bpm)	389 ± 8.5	392 ± 7.6	384 ± 6.5	403 ± 3.5

Values are means ± SEM

*p < 0.05 vs. sham/intact; ^†^*p* < 0.05 vs. ACF/intact

**Fig. 1:**
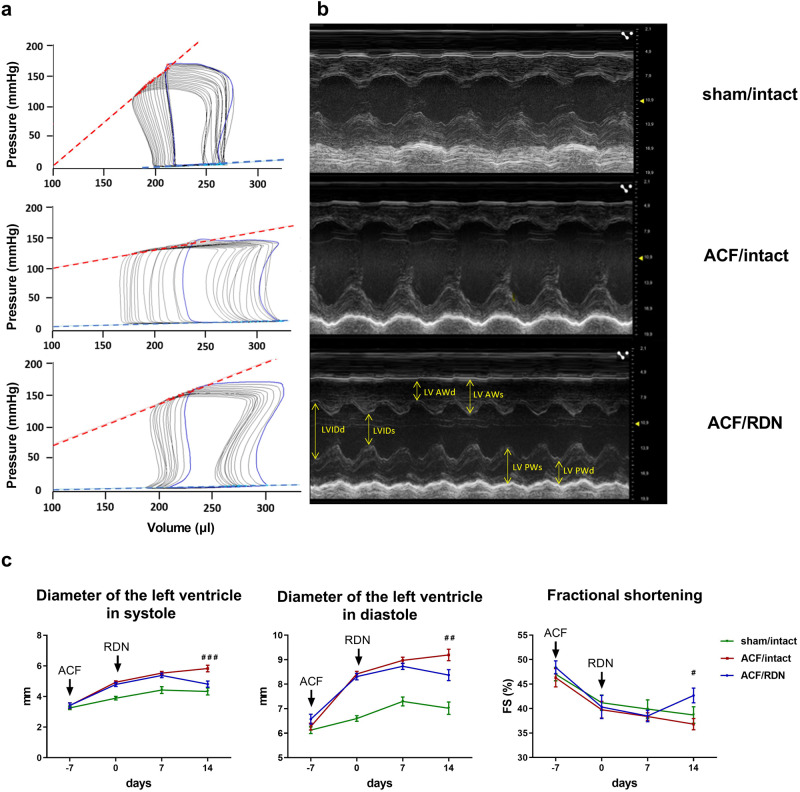
In vivo measurement of LV contractility and dimensions. **a** Representative pressure-volume loops from invasive pressure-volume analysis. Red line—end-systolic elastance (Ees), blue line—end-diastolic pressure-volume relationship (EDPVR). **b** Echocardiographic M mode images of parasternal long axis view. LV AWd left ventricular anterior wall thickness in diastole, LV AWs left ventricular anterior wall thickness in systole, LVIDd left ventricular internal diameter in diastole, LVIDs left ventricular internal diameter in systole, LV PWd left ventricular posterior wall thickness in diastole, LV PWs left ventricular posterior wall thickness in systole. **c** Diameter of left ventricle in systole (LVIDs) and diastole (LVIDd) measured during each week of experiment (3 weeks); FS fractional shortening. *N* = 10 in sham/intact, *N* = 19 in ACF/intact, *N* = 13 in ACF/RDN. ^###^*p* < 0.001; ^##^*p* < 0.01; ^#^*p* < 0.05, ACF/intact vs. ACF/RDN group, compared to the day 14

ACF had an impact on multiple organ weight parameters that are typically changed in HF, with no effect on the body weight or tibia length (not shown). ACF/intact rats had increased heart weight and LV weight. Similarly, compared to the sham group, ACF/intact rats had significantly increased weight of the left atrium (LA) and weight of the lungs, reflecting HF-related congestion. Compared to the sham/intact group, ACF/intact rats had significantly increased stroke volume, stroke work, and cardiac output. End-systolic and end-diastolic pressure (EDP) measured by PV analysis were increased in ACF/intact group compared to the sham/intact group, similar to echocardiographic measurements (Fig. 1b, c). Moreover, ACF rats had also a significant decrease in end-systolic pressure (143 ± 3.1 vs. 169 ± 3.7 mmHg, *p* < 0.05) compared to sham rats.

Compared to intact ACF, RDN significantly decreased heart weight, LA, LV weight, and congestion of the lungs and liver. RDN in ACF rats significantly decreased stroke work and normalized stroke volume and cardiac output. RDN in ACF rats also decreased dilatation of LV (Fig. 1b, c), which was shown as reduced LV end-systolic and end-diastolic volumes. We observed that ACF/RDN group had also reduced EDP (8.2 ± 0.69 vs. 12.7 ± 1.63 mmHg, *p* < 0.05) compared to ACF intact group. Heart rate was not affected by ACF or RDN in any groups.

### LV function and HF markers: the impact of ACF

**Fig. 2:**
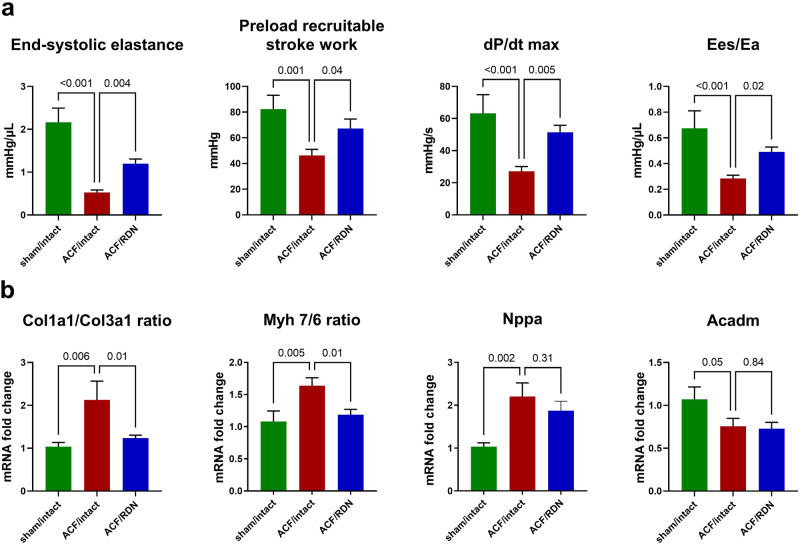
LV function and gene expression of selected HF markers and the impact of RDN. **a** Systolic function parameters measured by invasive PV analysis, Ees/Ea, ventricular-arterial coupling ratio. **b** Gene expression of markers of fibrosis—collagen I/III (Col1a1/Col3a1) ratio, myocardial stress—Myosin heavy chain 7/6 (Myh 7/6) ratio, natriuretic peptide A (Nppa) and mitochondrial fatty acid beta-oxidation pathway, acyl-CoA dehydrogenase medium chain (Acadm). *N* = 9 in sham/intact, *N* = 9 in ACF/intact, *N* = 10 in ACF/RDN

ACF/intact group had significantly decreased systolic function compared to the sham/intact group. ACF/intact group had also decreased end-systolic elastance (Ees) and preload recruitable stroke work (PRSW) compared to the sham/intact group. ACF/intact had also decreased ventricular-arterial coupling compared to sham/intact (Ees/Ea ratio, Fig. 2a).

ACF/intact group had extensive upregulation of markers of myocardial damage/remodeling compared to the sham/intact group. ACF/intact group had increased fibrotic marker collagen I/III (Col1a1/Col3a1) gene expression ratio. Similarly, maladaptive hypertrophy markers myosin heavy chain isotype ratio (Myh 7/6) and myocardial stress gene natriuretic peptide A (Nppa) were increased in ACF/intact group compared to the sham/intact group. ACF/intact group had a significantly decreased (*p* = 0.05) medium-chain acyl-Coa dehydrogenase (Acadm, Fig. 2b).

### Cardiac autonomic nervous system: the impact of ACF

**Fig. 3:**
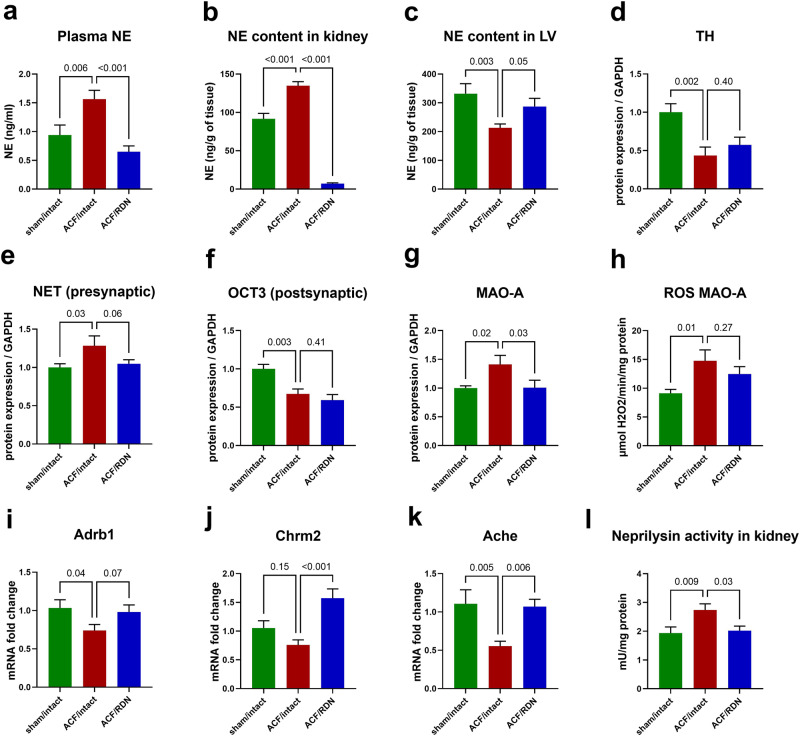
Impact of ACF and effects of RDN on selected parameters of sympathetic nervous system in left ventricle. **a** Plasma norepinephrine (NE). **b** NE content in kidney. **c** NE content in left ventricle (LV). **d** Biosynthesis of NE—protein expression of tyrosine hydroxylase (TH). **e** Preganglionic NE transport—protein expression of NE transporter (NET). **f** NE transport to cardiomyocyte—protein expression of organic cation transporter 3 (OCT3). **g** Degradation of NE—protein expression of monoamine oxidase A (MAO-A). **h** Production of reactive oxygen species (ROS) by MAO-A. **i** Gene expression of beta-1 adrenergic receptor (Adrb1). **j** Gene expression of choline muscarinic receptor type 2 (Chrm2). **k** Gene expression of acetylcholinesterase (Ache). **l** Neprilysin activity measured in kidney. *N* = 8 in sham/intact, *N* = 8 in ACF/intact, *N* = 8 in ACF/RDN

**Fig. 4:**
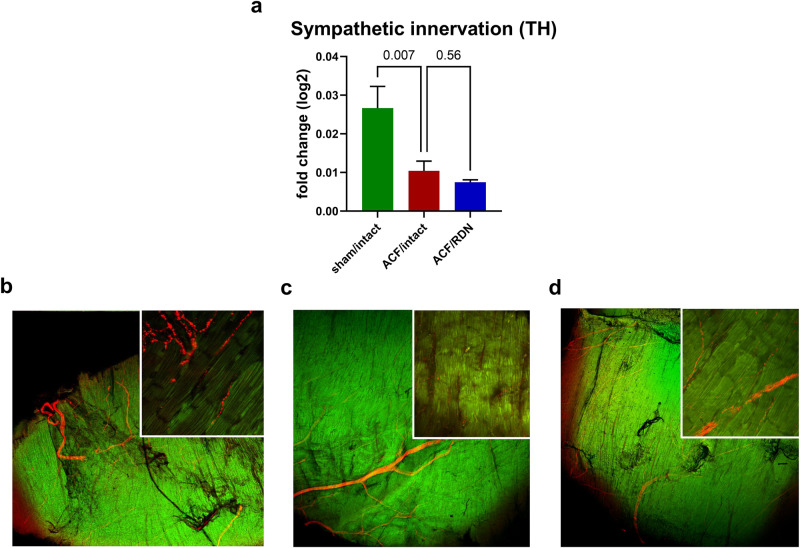
Results of immunohistochemical staining of tyrosine hydroxylase (TH, red color) in left ventricle. Zoom 25x in a smaller square embedded in an illustrative zoom 2x in a larger square. **a** Ratio of sympathetic nerves immunostained with TH antibody to the total area. **b** sham/intact. **c** ACF/intact. **d** ACF/RDN. *N* = 4 in sham/intact, *N* = 5 in ACF/intact, *N* = 4 in ACF/RDN

ACF rats had significantly increased NE levels in plasma and kidney (Fig. 3a, b), but depleted LV content of NE compared to the sham group (Fig. 3c). Correspondingly, we observed decreased LV protein expression of the key NE-synthetizing enzyme tyrosine hydroxylase (TH) in the LV (Fig. 3d) and diminished LV myocardial density of TH-positive sympathetic nerves (Fig. 4a, c, d). From proteins involved in the myocardial fate of NE, we observed an increased expression (*p* = 0.03) of presynaptic norepinephrine transporter (NET, responsible for synaptic NE reuptake, Fig. 3e) and significant decrease of organic cation transporter (OCT3, responsible for myocardial uptake of NE) in ACF compared to the sham/control group (Fig. 3f). MAO-A, NE-degrading enzyme was upregulated (Fig. 3g) and correspondingly, ROS generated by MAO-A (Fig. 3h) were increased in ACF LV, while gene expression of Adrb1 was downregulated compared to sham/intact group (Fig. 3i).

In parasympathetic cardiac signalization, ACF/intact rats had decreased acetylcholinesterase (Ache) and an unsignificant trend to decreased choline muscarinic receptor type 2 (Chrm2, Fig. 3j, k) in the LV compared to sham/intact.

ACF/intact rats displayed increased neprilysin activity in the kidney, compared to the sham/intact group (Fig. 3l).

### LV function and HF markers: the impact of RDN

RDN procedure significantly improved LV systolic function in ACF/RDN animals compared to the ACF/intact group. ACF/RDN group had increased Ees, PRSW, and Ees/Ea ratio compared to ACF/intact group (Fig. 2a). Peak LV pressure or effective arterial elastance (Ea) was not affected by RDN (2 ± 0.23 vs. 2.2 ± 0.22, *p* = 0.5, see Supplementary Information).

ACF/RDN group had less elevated markers of adverse myocardial remodeling compared to ACF/intact group—reduced gene expression of fibrotic markers (Col1a1/Col3a1 ratio), decreased the Myh 7/6 ratio compared to ACF/intact group, while Nppa gene expression was not significantly reduced. After RDN in the ACF group, we did not observe any changes in medium-chain fatty acids in gene expression of Acadm compared to ACF/intact rats (Fig. 2b).

### Cardiac autonomic nervous system in HF: the impact of RDN

RDN in ACF rats significantly reduced NE in the plasma and in the kidney (Fig. 3a, b). Despite we targeted the sympathetic nervous system in the kidney, we observed profound changes of sympathetic nerves in the heart—RDN led to increased NE levels in LV compared to ACF/intact rats (Fig. 3c).

In ACF/RDN group, we observed a numerically higher, but not significant increase in protein expression of TH (Fig. 3d). Sympathetic nerve density measured by TH staining was not significantly changed in ACF/RDN compared to ACF/intact (Fig. 4a, d). There was no difference in OCT3 (Fig. 3f), but strong trend to reduce protein expression of NET (presynaptic NE reuptake, *p* = 0.06, Fig. 3e) and significantly reduced MAO-A in ACF/RDN group, compared to ACF/intact rats (Fig. 3g). RDN/ACF rats displayed a trend to (*p* = 0.07) to higher gene expression of Adrb1 (Fig. 3i), and significantly increased gene expression of Chrm2 and Ache (Fig. 3j, k). We observed a significant positive correlation between gene expression of Adrb1 and TH among all groups (see Supplementary Information). RDN in ACF rats significantly reduced the activity of neprilysin in the kidney, compared to ACF/intact rats (Fig. 3l).

## Discussion

The principal findings of our study were that volume overload due to ACF leads to overt HF with congestion, accompanied by cardiac dysfunction, myocardial remodeling, reduced NE content and sympathetic nerve density in the heart, and enhanced sympathetic activation in the kidney. RDN procedure in our HF model reduced congestion and improved cardiac function, independently of changes in afterload, improved markers of cardiac remodeling, and partly restored cardiac NE levels and autonomic signaling. The study indicates that RDN has remote favorable intrinsic effects within the heart and it suggests that RDN could be a therapeutic approach not only for hypertension but also for HF.

### Impact of ACF on cardiac function

First, we confirmed that volume overload due to ACF leads to changes in the heart and in other organs consistent with overt HF, which was associated with intrinsic impairment of cardiac function by load-independent PV analysis [22, 35, 36, 45]. We validated our previous observation that the main NE-degrading enzyme—MAO-A is massively upregulated in failing ACF heart, which raised the question of how is cardiac sympathetic system disarranged in ACF [26]. Despite renal and circulating NE levels were massively increased in our HF model, NE levels were reduced in the failing myocardium, in agreement with other HF models, and in humans with advanced HF [5, 6, 46, 47]. Myocardial NE depletion occurred probably due to a combination of increased spillover from the sympathetic endings in the heart due to increased central sympathetic drive, and due to enhanced NE degradation by MAO-A.

Despite altered NE signaling in the failing heart cannot per se explain full cardiac dysfunction [48], it may contribute to abnormal cardiac performance. Increased cardiac NE spillover from sympathetic nerve endings leads to myocardial beta-1 adrenergic downregulation [7–9], diminished heart rate response, and cardiac performance during stress [49]. Increased myocardial NE catabolism by MAO-A, a major ROS-generating enzyme, may promote ROS-driven adverse cardiac remodeling [6]. We found evidence for all these mechanisms in our HF model, indicating that an intact cardiac sympathetic system is relevant for normal cardiac function.

In contrast with some [6, 50] but not all authors [5, 51], we did not find evidence for reduced synaptic NE reuptake in the failing myocardium as the cause of NE depletion. However, we did observe reduced sympathetic nerve density, which has also been found in other experimental HF models [5, 52, 53]. This reduction could result from inadequate sympathetic nerve growth, not paralleling extensive cardiomyocyte hypertrophy (a “dilution” effect), or from nerve damage, caused by high levels of ROS generated by MAO-A [7]. Additionally, the failing ACF hearts exhibited evidence of diminished parasympathetic signaling, suggesting that a vagal withdrawal also contributed to an autonomic imbalance in the failing heart.

### Impact of RDN on HF

Using load-independent PV analysis, our data convincingly demonstrate, for the first time, that RDN improves intrinsic myocardial indexes of contractility, such as Ees, PRSW, and Ees/Ea ratio, that were impaired by volume overload. This suggests that favorable myocardial effects of RDN in our HF model occurred beyond simple reduction of cardiac afterload. Reduction in cardiac output and stroke volume in ACF by RDN is considered beneficial and reflects the reduction of hypertrophy and dilation. The restitution of load-independent contractility was also accompanied by direct evidence of more favorable myocardial remodeling, as indicated by less abnormal gene expression of Myh 7/6 ratio and collagen I/III ratio in the ACF heart after RDN. The improvements in cardiac function and remodeling were complemented by the marked effects of RDN on the cardiac autonomic nervous system.

Notably, RDN in ACF animals reduced circulating NE, but it increased abnormally low NE levels in the LV. The increase of myocardial NE content was not explainable by increased growth of sympathetic nerves (as TH gene expression and nerve densities were similar), or presynaptic or postsynaptic transport by NET or OCT3. Increased myocardial NE content may be due to attenuated central sympathetic drive by RDN, with less NE being released from nerve endings. It is known from previous studies, that RDN eliminates not only efferent sympathetic nerves but also afferent, centrally projecting nerve fibers, which regulate the central sympathetic drive toward the heart [1, 3, 54], and these central effects of RDN may be actually dominant. Experimental denervation of the stellate ganglion and renal afferent denervation had a similar cardiorenal protective effect [55]. Based on the observed effects of RDN on NE plasmatic and cardiac levels in the ACF model, we propose that the mechanism leading to increased myocardial NE content may be in reduced cardiac spillover due to diminished central sympathetic drive toward the heart, which was not measured in our study but was confirmed by direct neural recordings in a sheep HF model [56]. Myocardial MAO-A expression, which was also reduced after RDN, may be responsive to diminished NE exposure and this decrease in MAO-A may further beget less NE degradation [6]. Follow-up studies with measurements of myocardial NE kinetics and with measurements of systemic sympathetic nerve activity would be necessary to confirm our assumptions.

Besides the strong effect of RDN on the cardiac sympathetic system, RDN may influence parasympathetic signaling, as evidenced by increased gene expression of Chrm2 and Ache in ACF. A catheter-based RDN in ovine HF supports this suggestion that RDN is increasing cardiac parasympathetic nerve activity and controls heart rate, however, further comprehensive investigations are required to fully understand the effect of RDN on the cardiac parasympathetic nervous system [56, 57].

The improvements in cardiac structure and function induced by RDN are more likely to explain improved survival in the ACF model, than the direct effects of RDN on renal hemodynamics. This was shown by our previous study, where RDN improved survival rate, but had no effect on reduced renal blood flow or exaggerated renal vascular responsiveness to angiotensin II [19]. Yet, the kidneys can participate in the beneficial effect of RDN by less pronounced neurohumoral activation in ACF after RDN. Besides reducing renal NE content and spillover, RDN reduces renal neprilysin activity, thus leading to less degradation of cardioprotective natriuretic peptides [2, 43]. RDN has therefore similar, or even greater, effects as neprilysin inhibitor sacubitril [43].

### Effect of RDN in other HF models and clinical implications

Experimental studies of RDN in other HF models, using less accurate methodology, also indicated, that cardioprotective effects of RDN may be mediated beyond pressure reduction [19, 24]. Besides the effects of RDN on cardiac hypertrophy and congestion, RDN improved myocardial function, ventricular and atrial fibrosis in the ischemia-reperfusion swine model [58], and in rabbits with rapid ventricular pacing [16]. RDN also improved survival, decreased sympathetic nerve activity and catecholamine spillover, reduced fibrosis, and improved LV function (assessed by LV ejection fraction) in myocardial infarction-induced HF in dogs, or in rats [2, 32, 43, 59]. None of these studies used precise load-independent LV function assessment, in contrast to our study.

The specific clinical implication of RDN lies in its potential to modulate the sympathetic nervous system [12, 60, 61]. Sympathetic overactivity is associated with conditions such as HF, resistant hypertension, and chronic kidney disease. RDN aims to disrupt the excessive sympathetic nerve activity by selectively ablating or modulating the renal sympathetic nerves, potentially leading to a reduction in sympathetic outflow and restoration of NE signaling in the heart [18, 62], attenuation of neurohumoral activation, and normalization of cardiac autonomic control. This modulation of sympathetic activity by RDN may help manage HF, improve cardiac function, and potentially improve outcomes [3, 10, 56]. Despite RDN affects multiple targets in HF, it is likely still relatively organ-selective, not causing systemic hypotension, in contrast to an unselective drug-induced central inhibition of sympathetic outflow, that was associated with worse outcomes in HF [20]. The absence of the hypotensive effect of RDN in HF models is also an important aspect, because hypotension complicates the management of more advanced phases of HF and often represents a critical limit for more aggressive pharmacotherapy.

Our study has some limitations. To accelerate the onset of cardiac dysfunction, which in normotensive strains may take 15–20 weeks [30, 63], we utilized the hypertensive TGR strain, where HF develops earlier and myocardial changes may be more pronounced than in normotensive strains. Due to high pressure and volume overload in ACF, an increased volume is ejected into the arteries, and consequently, there is a decrease in vascular resistance, elasticity, and peripheral vascular resistance [64]. Thus, Ea is already significantly reduced in the early stages of the ACF model (Supplementary Fig. 1), and RDN probably did not have the capacity to reduce it even lower. We used unselective chemical and mechanical ablation of the renal nerves which may differ from RDN by radiofrequency energy. The durability of RDN effects and potential reinnervation was not studied. We did not directly measure the central sympathetic nerve drive or NE spillover. Based on RDN-induced changes in the cardiac autonomic nervous system it could be suggested that systemic sympathetic nerve activity was decreased after RDN, however, we did not evaluate any other markers of systemic sympathetic nerve activity. Because we used NE kidney levels as a marker of success of RDN, we cannot use them as a marker of reduced systemic sympathetic nerve activity in denervated rats. Unfortunately, we cannot quantify and localize which part of myocardial MAO-A came from sympathetic neurons or from cardiomyocytes.

In conclusion, our results showed that RDN improved LV contractility and function independently of cardiac loading, attenuated abnormal cardiac remodeling, restored cardiac NE levels and cardiac autonomic signaling in HF, induced by chronic volume overload. These changes likely explain previously observed improvement of survival after RDN in this model [19]. The results suggest that RDN has remote favorable intrinsic effects within the heart and RDN could be a useful therapeutic strategy in HF.

## Supplementary information

Supplementary Figure 1

Supplementary Figure 2

Supplementary Materials
