# Supplementary figures and images for: Renal denervation improves cardiac function independently of afterload and restores myocardial norepinephrine levels in a rodent heart failure model
Source: Hypertens Res. 2024 Feb 2;47(10):2718–30. doi: 10.1038/s41440-024-01580-3 (PMC11456508; doi:10.1038/s41440-024-01580-3)

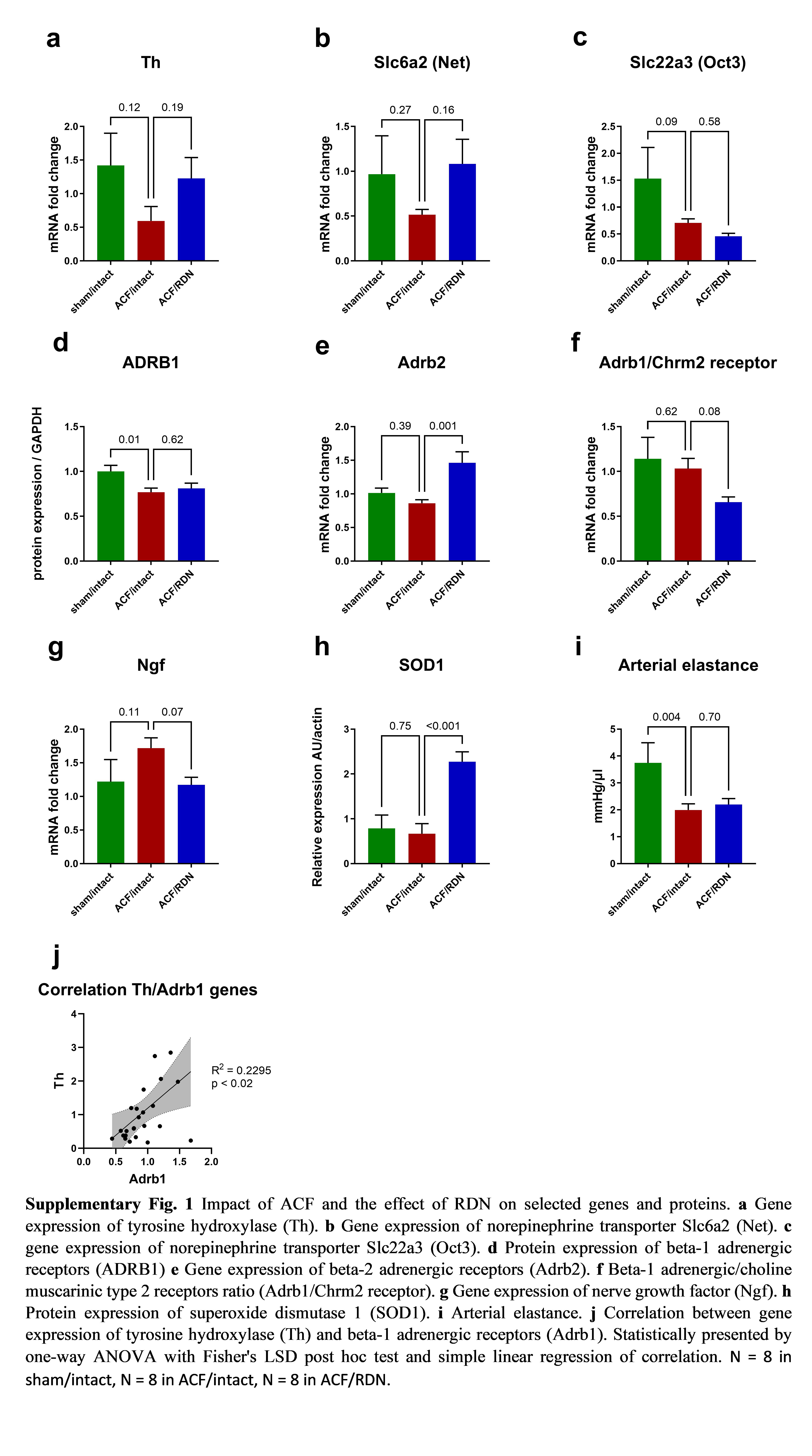

Supplement: Supplementary file 1 — Supplementary Figure 1 [file 41440_2024_1580_MOESM1_ESM.jpg]

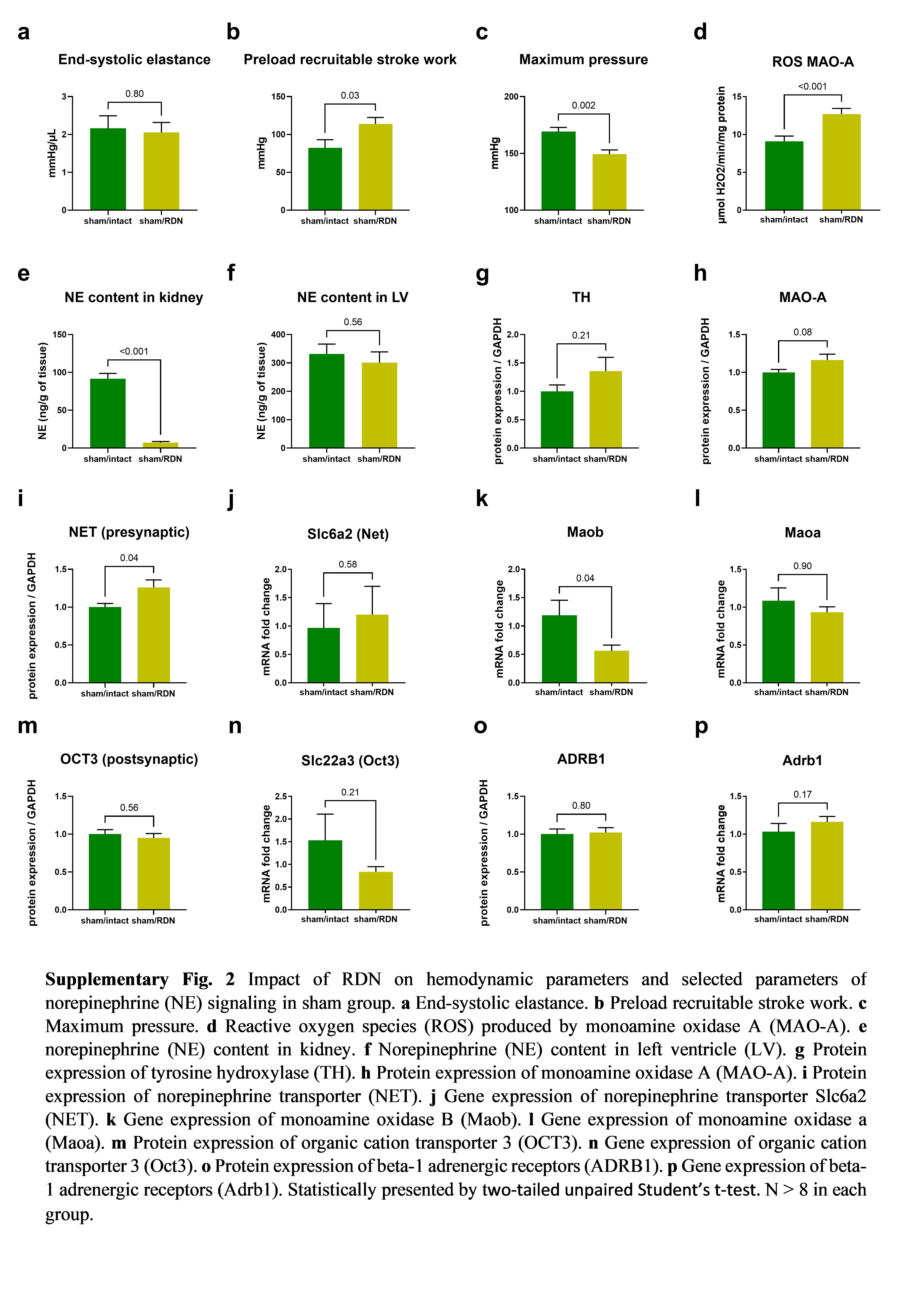

Supplement: Supplementary file 2 — Supplementary Figure 2 [file 41440_2024_1580_MOESM2_ESM.jpg]
