# Supplementary material for: Renal denervation improves cardiac function independently of afterload and restores myocardial norepinephrine levels in a rodent heart failure model
Source: Hypertens Res. 2024 Feb 2;47(10):2718–30. doi: 10.1038/s41440-024-01580-3 (PMC11456508; doi:10.1038/s41440-024-01580-3)
Supplement: Supplementary file 3 — Supplementary Materials [file 41440_2024_1580_MOESM3_ESM.docx]

**Supplementary Materials**

**Methods**

**Animals**

The study was performed in accordance with relevant regulations and was approved by the Animal Ethic Committee of IKEM and by the Ministry of Health of the Czech Republic (#12468/2021-5/OVZ).

All animals were housed in transparent plastic cages for four animals under stable 12 hours light to 12 hours dark conditions at controlled room with temperature (22 ± 1°C) and humidity (40%). Rats had free access to tap water and were fed a normal protein diet (19-21% protein, 0.45% NaCl) manufactured by SEMED (Prague, Czech Republic). Experiments were performed in male Ren-2 transgenic hypertensive rats (TGR) at initial age of 8 weeks. All animals used in this study were bred at the Center for Experimental Medicine of this Institute from stock animals supplied by the Max Delbrück Center for Molecular Medicine, Berlin, Germany. Heterozygous TGR were generated by breeding female homozygous HanSD rats with male homozygous TGR as described in original study (1). 8-week-old Ren-2 transgenic male rats (model of angiotensin II-dependent hypertension) underwent ACF surgery to induce volume overload. After one week, animals underwent bilateral RDN (mechanical and chemical by topical phenol application) from laparotomy. Two weeks after RDN, the functions of both ventricles were measured by simultaneous biventricular pressure-volume analysis, and the animals were examined using echocardiography each week. Animals were killed by thiopental sodium i.p. overdose (in the case of in vivo measurements of echocardiography and pressure-volume analysis), or by decapitation (hormonal and molecular analyses).

**Creation of aortocaval fistula**

The animals were anaesthetized using ketamine and midazolam applied intraperitoneally (Calypsol, Gedeon Richter, Hungary, 160 mg/kg and Midazolam, Kalcex, Latvia, 160 mg/kg). The aortocaval fistula was created as described previously and this procedure is routinely performed in our laboratory (2–4). The abdominal aorta was pierced into the inferior vena cava by an 18-gauge needle (1.2 mm in diameter) and bulldog vascular clamp was placed across the caudal aorta. The needle was removed, and an acrylamide tissue glue (Histoacryl, B.Braun AG, Germany) was applied at the puncture point. After sealing the wound with tissue glue, the clamp was removed and the aortocaval shunt was checked visually by vena cava pulsation. The sham-operated rats underwent opening and closing of the abdominal cavity, without the aortocaval fistula procedure. After the surgery, meloxicam analgesia (1–2 mg/kg/ day SC) was given for 2–3 days.

**Renal denervation**

Bilateral RDN procedure was performed one week after ACF surgery. Animals were anaesthetized using ketamine/midazolam, both kidneys were approached through a midline abdominal incision, both renal arteries and veins were separated from the connective tissue and all the visible nerve fibers were stripped of the arteries and cut close to the hilus. Phenol (10% solution in ethanol) was dripped onto both renal arteries in order to destroy any remaining nerve fibers. The adjacent tissues were carefully protected, from exposure to the phenol solution and disruption of the major lymphatic vessels in the area was avoided. Intact animals underwent laparotomy and retraction of the abdominal organs, but renal vessels were not isolated or painted with phenol solution and the renal nerves were not severed; instead, the intraabdominal area was coated with a 0.9% NaCl solution. This experimental procedure of RDN, was described and fully effective in previous studies (3, 5–7).

**Echocardiography**

Echocardiographic examination was performed in the day of surgery before creation of aorto-caval fistula/sham. Second examination was performed after one week before renal denervation/intact surgery and once per week during next two weeks until end of experiment. Prior the echocardiographic examination, the animals were anesthetized with 4% isoflurane (IsoVet®, Piramal Healthcare, UK). During the image acquisition, the rats were maintained under isoflurane anesthesia (1.5-2%, Combi-vet® system, Rothacher Medical GmbH, Heitenried, Switzerland), and fixed in the supine position. B-Mode and M-Mode images were recorded in parasternal long and short axis view and used for measurements of dimensions of LV internal diameter, anterior and posterior walls. Echocardiographic examination was done by Vevo® 2100 Imaging System with the MS250S transducer (13–24 MHz), and evaluated in VevoLab (v3.2.0, FUJIFILM VisualSonics, Inc., Toronto, ON, Canada).

**Pressure-volume analysis**

Rats were anesthetized with long-term stable anesthesia often used for pressure-volume analysis (thiopental, 50 mg/kg, IP, VAUB Pharma a.s., Roztoky, CZ). Before pressure-volume analysis, echocardiography was performed as described above. Rats were intubated with a plastic cannula to ensure chest relaxation during the whole operation and were artificially ventilated through the procedure (Ugo Basile, Gemonio, IT) with the rat's hind paw attached to the oximeter flap for continuous O2 monitoring (Nonin Model 2500A VET, Nonin Medical, USA). Ventilation settings were based on the animal’s weight using the following formulas: respiration rate (RR, min^−1^) = 53.5 × M^−0.26^; tidal volume (V_t_, ml) = 6.2 M^1.01^ (M = animal mass/kg). The left jugular vein was cannulated with saline for securing central venous access. A balloon catheter (LeMaitre Single Lumen Embolectomy Catheter, 2F, Burlington, MA, USA) was inserted under ultrasonic control via the right jugular vein to the vena cava inferior, below the diaphragm to maintain the best position for preload reduction (Ref Miklovic JPP, in press). Just before the pressure-volume measurement of the left ventricle, the conductance and pressure signals of the Millar pressure-volume catheter (Millar, 2F, Houston, TX, USA) were calibrated using MPVS software (V2.2, Millar, Houston, TX, USA) according to the manufacturer's instructions. Functions of the left ventricle were invasively assessed by a pressure-volume catheter introduced into the left ventricle via the right carotid artery as described in previous studies (8–10). For basal measurements, pancuronium (1 mg/kg, IV, Inresa Arznemittel, Freiburg, DE) was administered through the cannulated left jugular vein and rinsed with a bolus of saline to reduce noisiness in the signal caused by breathing. For effective determination of cardiac functions during hemodynamic transients, the preload reductions were performed by slowly inflating the balloon catheter with aqua pour injection. Volume signal was calibrated by cuvette calibration unit of known volumes fulfilled with heparinized warm blood taken from left ventricle after the experiment (Millar, Houston, TX, USA). Data from pressure-volume loops were captured and analyzed in LabChart Pro software (ADInstruments, Bella Vista, NSW, Australia) as discussed in detail in our previous study (Miklovic ... JPP).

**Gene expression analysis**

Prior to the RNA extraction, all surfaces, racks and devices were decontaminated with RNAse Away (Molecular Researcher Centre, Inc., Cincinnati, OH, USA) for 20-30 min. Homogenization beads were also soaked in RNAse Away and then washed in 99.9% ethanol. Left ventricle of the heart was homogenized in 1 ml of RNAzol® RT (#RN190; Molecular Research Center, Inc., Cincinnati, USA) with glass beads (2 mm) in the mixture mill (Retch mixture mill MM 400, Retsch GmbH, Haan, Germany) for 10 min (30/s). Then the samples were incubated for 10 min at room temperature (rt) to complete the dissociation of nucleoprotein complexes. Thereafter 0.4 ml of RNA free water was added to each sample, mixed vigorously for 15 seconds and then incubated again for 15 min., rt to allow the precipitation of proteins. After the precipitation the samples were centrifuged (12000 g, 15 minutes, 4°C). Then 0.75 ml of the supernatant containing the soluble RNA was carefully transferred to a new tube. Only around 75% of the supernatant was collected to make sure that the semisolid pellet containing DNA, proteins and most of the polysaccharides remained untouched. Thereafter the RNA was precipitated with 0.4 ml of 75% ethanol and then the tubes were gently mixed for 1 min. The samples were incubated for 15 min., rt and centrifuged (12000 g, 20 min, 4°C). RNA precipitate formed a white pellet at the bottom of the tube and the supernatant was gently discarded. Then the pellet was washed twice with 1 ml of 75% ethanol, followed by brief vortexing and centrifugation (7500 g, 5 min, 4°C). The pellet was dried in a thermoblock for around 10 min at 55°C (or slightly longer until all of the remaining ethanol was evaporated). The pellet was dissolved in RNA free water (50 μl) and then the closed tubes were incubated again in the thermoblock for 10 min. to provide the complete dissolution of the pellet. Then the samples were incubated for 1 hour in room temperature and stored at -80°C until analysis.

The concentration and purity of the extracted RNA samples were measured with microvolume spectrophotometer (DeNovix Inc., Wilmington, USA). Then the samples were diluted to yield the concentration of 1 µg per 5 µl of RNA free water, *i.e.* the final concentration of RNA was 2 µg in 20 µl of the reaction mixture for reverse transcription. Bio-Rad C1000 Thermal cycler (Bio-Rad s.r.o., Prague, Czech Republic) was used with High Capacity cDNA Reverse Transcription Kit (#4368813; Applied Biosystems, Foster City, CA, USA). The reaction parameters and conditions were set according to the manufacturer’s recommendations.

20 µl of each transcribed cDNA sample was diluted with 35 µl of DEPC-treated water and mixed with 55 µl of TaqMan^@^ Fast Advanced Master Mix (#4444556; Applied Biosystems, Foster City, CA, USA) and then the whole solution was transferred to a predesingned TaqMan^@^ Array Card. The cards were centrifuged twice (1200 RPM, 1 min, 4°C) and sealed. The quantification real time PCR was done on ViiA™ 7 Real-time PCR system (Applied Biosystems, Foster City, CA, USA) according to the manufacturer’s recommendation. The measurement of multiple mRNA expressions was performed in accordance with the manufacturer’s instructions (384-well microfluidics TaqMan array cards; custom setting of selected genes; Applied Biosystems, Foster City, CA, USA).

In all experiments, relative gene expression was calculated by the *2-∆∆Ct* method, which is the most frequently used for such experiments (11–13). This method directly uses the Ct (threshold cycle) information generated from a qPCR system. *Ct* is the cycle number where the fluorescence generated by the PCR produce is distinguishable from the background noise. To calculate relative gene expression in target and reference samples, was used a housekeeping gene GAPDH as the normalizer (11, 14, 15).

The expression of mRNA of selected genes was related to a control group rats. Final results were expressed as the n-fold difference in gene expression of mRNA of target genes between experimental and control group.

**Immunohistochemistry**

Dissected basal halves of free walls of the RV and LV were whole-mount stained with anti-tyrosine hydroxylase primary antibody (1:500, rabbit AB152, Merck Millipore) visualized with Cy5 coupled goat-anti-rabbit secondary antibody (1:200, Jackson Immuno Research) as described (16). Incubation times were prolonged (blocking 24 hours in normal goat serum, 1:10, primary antibody 48 h, washing 12 h, secondary antibody 24 h, all at rt with continuous gentle rocking), and Triton-X concentration was increased at 0.1 %.

After staining and thorough rinsing in PBS, the samples pinned epicardial side up to the bottom of a deep Petri dish covered with Sylgard and cleared in Scale2 for at least 48 h (17). The samples were then examined on a confocal Olympus BX61 system (FluoView 1000) using a 2x, 0.14 NA dry objective (overview picture) followed by 25x ScaleView objective (NA 1.0). Three confocal stacks spanning at least 50-micron depth were collected from different location at 1-micron z-step. For analysis, substacks of 10 µm thickness were selected from the subepicardial region, and maximum intensity projections of green channel (488 nm excitation, detecting tissue autofluorescence derived mostly from the myocytes) and far-red channel (635 nm excitation, specific fluorescence of anti-tyrosine hydroxylase immunostaining) were created in ImageJ. Percentage of red to red + green channel was then calculated after signal thresholding in ImageJ.

**Myocardial ROS production by monoamine oxidase A**

Myocardial hydrogen peroxide production by monoamine oxidase A (MAO-A) was determined fluorometrically measuring the oxidation of Amplex Red reagent (Thermo Fisher Scientific) coupled to the enzymatic reduction of H2O2 by horseradish peroxidase. Briefly, 10% homogenates were prepared in ice-cold KCl-based media (120 mM KCl, 3 mM HEPES, 5 mM KH2PO4, 3 mM MgSO4, and 1 mM EGTA, pH 7.2) from frozen free wall of LV using zirconium oxide grinding balls (3 min, 30 Hz), Retsch MM 400 mixer mill (Retsch, Haan, Germany) and filtered through a fine mesh. Protein concentration was estimated by the BCA method. An aliquot of the homogenate was saved for SDS-PAGE analyses. The assay was performed using 0.15 mg/mL of protein in KCl based media with or without the MAO-A substrate tyramine (50uM) or its specific inhibitor clorgylin (1 μM), in the presence of 50μM Amplex Red and 1 U/mL HPR. A standard curve of 1 to 10 μM H2O2 was established in every plate to calibrate the signal to nmol produced. The increase in the fluorescence was recorded for 30 minutes at 37°C with an Infinite M200 plate reader (Tecan Group Ltd., Männedorf, Switzerland) at 544/590 nm.

**Western blot analysis**

The heart protein expression of solute carrier family 22 member 3 (SLC22A3), also known as organic cation transporter 3 (OCT3), β1-AR, TH, MAO-A and NET were measured by western blot analysis. Briefly, LV tissue (free wall) was homogenized in 1:3 (tissue weight:volume) in ice–cold RIPA lysis buffer containing 50 mM Tris–HCl pH 7.4, 150 mM NaCl, 1% NP-40, 0.25% deoxycholic acid, 1 mM EDTA; supplemented with protease inhibitor cocktail (Sigma-Aldrich, St. Louis, MO, USA) using mixer mill MM400, kept on ice for 20 min and centrifuged twice - at 10.000 g for 10 min at 4°C and at 20.000 g for 10 min at 4°C. Protein concentration in the supernatant was measured using Pierce BCA protein assay (Thermo Fisher Scientific, Waltham, MA, USA). In total 80 μg of protein was separated by sodium dodecyl sulfate polyacrylamide gel electrophoresis (SDS-PAGE) and transferred onto the polyvinyl difluoride (PVDF) membrane in transfer buffer at 100 V for 1.5 hours. Membranes were blocked with 2,5% BSA/5% non-fat dry milk in TRIS buffered saline with Tween20 (TBS-T) for 1 hour at room temperature. After washing with TBS-T, the membranes were incubated with primary antibodies overnight at 4°C. The antibody dilutions were as follows:

anti-SLC22A3 (OCT3), 1:250, Invitrogen (PA5-39322; Waltham, Massachusetts, USA);

anti-β1-ADR, 1:400, Invitrogen (PA1-049; Waltham, Massachusetts, USA);

anti-TH, 1:200, Invitrogen (MA1-24654; Waltham, Massachusetts, USA);

anti-MAO-A, 1:5 000, Abcam (ab126751; Cambridge, UK);

anti-NET, 1:100, Invitrogen (PA5-89680; Waltham, Massachusetts, USA);

anti-GAPDH-HRP, 1:500, Invitrogen (31461; Waltham, Massachusetts, USA).

After overnight incubation and washing, the membranes were incubated with horseradish peroxidase-conjugated secondary antibody for 1 hour at room temperature. After last washing, the immunoblots were exposed to SuperSignal West Dura Substrate (Thermo Scientific, Rockford, IL, USA) for chemiluminiscent detection. Relative densitometry was determined using ImageJ software (NIH, Bethesda, MD, USA). All protein data was normalized to the housekeeping protein GAPDH. The protein expression was related to a compared control group. Final results were expressed as the n-fold difference in target protein expression between experimental and control group.

**Neprilysin (NEP) activity in kidney tissue**

NEP activity was assessed in whole kidney samples as described before (20, 55). Briefly, kidney tissue was homogenized in 1:4 (tissue weight:volume) in ice-cold NEP Assay buffer supplemented with protease inhibitors aprotinin and phenylmethylsulfonyl fluoride using mixer mill MM400, kept on ice for 10 min, centrifuged at 12.000 g for 10 min at 4°C and supernatant was collected. NEP activity was measured by a fluorometric assay (K487-100; BioVision, Milpitas, CA, USA). NEP activity kit utilizes the ability of an active NEP to cleave a synthetic substrate (Abz-based peptide) to release a free fluorophore. The release Abz can be easily quantified using a fluorescence microplate reader. One unit of NEP activity is the amount of enzyme that catalyzes the release of 1 μmol of Abz per min from the substrate under the assay conditions at 37 °C.

**The measurement of norepinephrine (NE) in kidney and heart tissue**

Norepinephrine (NE) was measured in kidney cortex and LV heart samples. Briefly, wet tissue samples were weighted and homogenized in 1:3 (tissue weight:volume) in 0,05M PB (pH 7.4) supplemented with protease inhibitor cocktail (Sigma-Aldrich, St. Louis, MO, USA) and ascorbic acid using mixer mill MM400, centrifuged twice - at 3.000 g for 10 min at 4°C and at 10.000 g for 10 min at 4°C, supernatant was collected. NE concentration was measured by a solid phase enzyme-linked immunosorbent assay based on the sandwich principle, using commercially available ELISA kit (RE59261; IBL International, Hamburg, Germany).

**Statistical analysis**

Statistical analysis of the data was performed using Graph-Pad Prism software v9.4.1 (Graph Pad Software, San Diego, CA, USA). All results are presented as the mean ± standard error of the mean. The data were analyzed using one-way ANOVA (organ weights, core hemodynamic parameters, gene and protein expressions) with Fisher's LSD post hoc test or two-way ANOVA (echocardiography results) followed by Tukey’s post hoc multiple comparison test. The values of p below 0.05 were considered as statistically significant. For better clarity, the impact of RDN on complex parameters of cardiac structure and function in sham rats is presented separately, assessed by two-tailed unpaired Student’s t-test in comparison to a relevant comparator group (sham/intact, see Supplementary information). The values of p below 0.05 were considered as statistically significant.
